# Simultaneous detection of lysine metabolites by a single LC–MS/MS method: monitoring lysine degradation in mouse plasma

**DOI:** 10.1186/s40064-016-1809-1

**Published:** 2016-02-25

**Authors:** Izabella A. Pena, Lygia A. Marques, Angelo B. A. Laranjeira, José A. Yunes, Marcos N. Eberlin, Paulo Arruda

**Affiliations:** Centro de Biologia Molecular e Engenharia Genética, Universidade Estadual de Campinas (UNICAMP), 13083-875 Campinas, SP Brazil; Thomson Mass Spectrometry Laboratory, Universidade Estadual de Campinas (UNICAMP), Campinas, SP 13083-861 Brazil; Centro Infantil Boldrini, Universidade Estadual de Campinas (UNICAMP), Campinas, SP 13083-210 Brazil; Departamento de Genética Médica, Faculdade de Ciências Médicas, Universidade Estadual de Campinas (UNICAMP), Campinas, SP 13083-887 Brazil; Departamento de Genética e Evolução, Instituto de Biologia, Universidade Estadual de Campinas (UNICAMP), Campinas, SP 13083-970 Brazil

**Keywords:** Lysine catabolism, Saccharopine, Amino acid, Mass spectrometry, Pipecolic acid

## Abstract

Detection and quantification of lysine degradation metabolites in plasma is necessary for the diagnosis and follow-up of diseases such as pyridoxine-dependent epilepsy. The principal metabolites involved in the disease are related to the first steps of lysine oxidation, either through the saccharopine or the pipecolate pathways. Currently, there are three different analytical methods used to assess the content of these metabolites in urine and plasma, but they require different sample preparations and analytical equipment. Here, we describe a protocol that calls for a simple sample preparation and uses liquid chromatography tandem mass spectrometry (LC–MS/MS) that allows simultaneous detection and quantification of underivatized l-saccharopine, l-aminoadipic acid, l-pipecolic acid, piperideine-6-carboxylate, l-glutamic acid, and pyridoxal-5-phosphate in plasma samples. To validate the method we analyzed the time course degradation after intraperitoneal injection of l-lysine in C57BL/6/J mice. We observed that the degradation of lysine through the saccharopine pathway reached a maximum within the first 2 h. At this time point there was an increase in the levels of the metabolites saccharopine, aminoadipic acid, and pipecolic acid by 3-, 24- and 3.4-fold, respectively, compared to time zero levels. These metabolites returned to basal levels after 4–6 h. In conclusion, we have developed a LC–MS/MS approach, which allows simultaneous analysis of lysine degradation metabolites without the need for derivatization.

**Electronic supplementary material:**

The online version of this article (doi:10.1186/s40064-016-1809-1) contains supplementary material, which is available to authorized users.

## Background

**Fig. 1:**
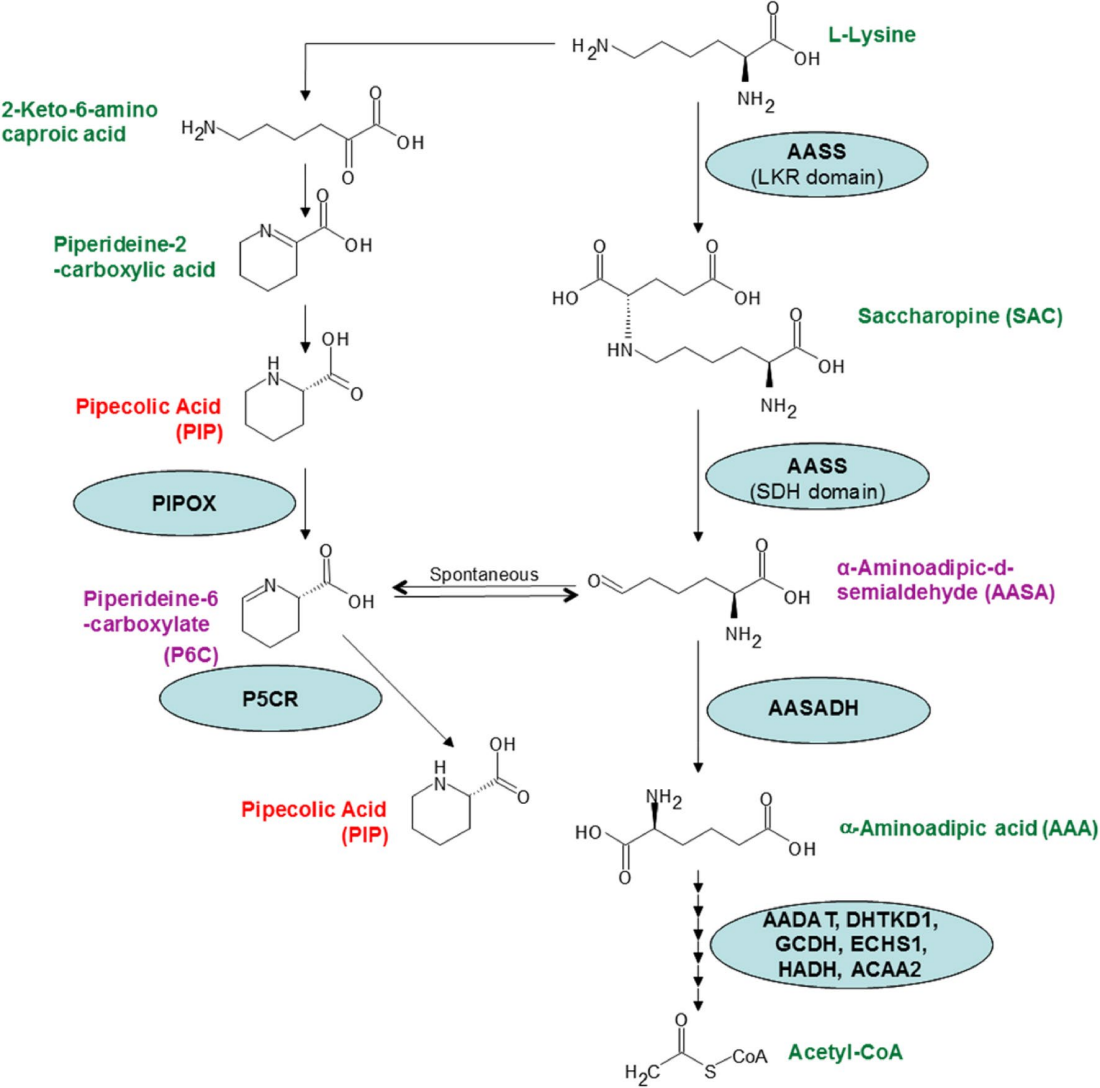
Schematic representation of lysine degradation pathways in mammals. Lysine can be degraded via pipecolate and saccharopine pathways. In the pipecolate pathway, that is believed to operate mostly in the brain, lysine is deaminated at the alpha nitrogen, and the resulted pipecolic acid is converted to P6C by pipecolate oxidase (PIPOX). In the saccharopine pathway that has been demonstrated in several tissues, lysine is deaminated at the epsilon nitrogen. In the first reaction step, catalyzed by the lysine-ketoglutarate reductase (LKR) domain of the aminoadipic semialdehyde synthase (AASS), lysine is condensed with α-ketoglutarate to form saccharopine. Than the saccharopine dehydrogenase (SDH) domain of AASS hydrolyse saccharopine into glutamic acid and α-aminoadipic-δ-semialdehyde (AASA). AASA is then oxidized to form aminoadipic acid (AAA) by the enzyme aminoadipic semialdehyde dehydrogenase (AASADH). AASA is in equilibrium with its cyclic form P6C. P6C can be used as substrate by the enzyme piperideine-5-carboxilic reductase (P5CR). AAA than proceeds to several enzymatic steps catalyzed by the enzymes AADAT (aminoadipate aminotransferase), DHTKD1 (dehydrogenase E1 and transketolase domain containing 1), GCDH (glutaryl-CoA dehydrogenase), ECHS1 (enoyl-CoA hydratase, short chain, 1), HADH (Hydroxyacyl-coenzyme A dehydrogenase) and ACAA2 (acetyl-CoA acyltransferase 2) to form acetyl-CoA. Mutations in the genes encoding AASADH and GCDH lead to PDE and glutaric aciduria type 1 disease, respectively

Lysine oxidation in mammals is essential for regulating the free levels of this amino acid, for the balance of nitrogen and conversion to ketone bodies. The saccharopine pathway is considered the main route for irreversible degradation of lysine in higher eukaryotes (Carson et al. 1968; Blemings et al. 1994; Arruda et al. 2000). The first step of this pathway is catalyzed by lysine-ketoglutarate reductase (LKR), which condenses lysine and α-ketoglutaric acid to form saccharopine (SAC) (Arruda et al. 2000). The next step is catalyzed by saccharopine dehydrogenase (SDH), which hydrolyses saccharopine into glutamic acid (GLU) and α-aminoadipic-δ-semialdehyde (AASA) (Fig. 1). In animals and plants, the activities of LKR and SDH belong to distinct domains of a bifunctional polypeptide called aminoadipic semialdehyde synthase (AASS) that is encoded by the gene *aass* (Markovitz et al. 1984; Goncalves-Butruille et al. 1996; Papes et al. 1999). An additional pathway for lysine degradation exists in humans, the pipecolate pathway (Fig. 1) (Chang 1978; Hallen et al. 2013). The saccharopine and pipecolate pathways differ in the way lysine is oxidized and which nitrogen group is removed to form AASA. In the pipecolate pathway, lysine degradation occurs via α-deamination whereas in the saccharopine pathway the reaction involves a ε-deamination. In both cases, lysine is deaminated and oxidized to produce AASA, which spontaneously cyclizes into piperideine-6-carboxylate (P6C). Both of these molecules function as substrates for the enzyme aminoadipic semialdehyde dehydrogenase (AASADH), in a rapid reaction that gives rise to aminoadipic acid (AAA) (Fig. 1).

Metabolites derived from lysine oxidation have sgnificant implications in the pathophysiology of specific diseases such as pyridoxine-dependent epilepsy (PDE). PDE is caused by a mutation in the *aldh7a1* gene that abolishes the AASADH activity and patients accumulate high levels of AASA/P6C and pipecolic acid (PIP) (Mills et al. 2006). Although there is a consensus that the saccharopine pathway plays a significant role in lysine catabolism, especially in liver and kidney (Higashino et al. 1971; Blemings et al. 1994; Papes, et al. 2001), recent reports have suggested that this pathway is virtually absent in the brain. Instead, the pipecolate pathway may be predominant in cerebral tissues, suggesting that this could be the target of choice for development of new therapeutic interventions (Sauer et al. 2011; Hallen et al. 2013; Posset et al. 2015). However, most of the experiments conducted to verify the levels of saccharopine or the absence of AASS activity in cerebral tissues were performed in mice without lysine supplementation and/or after several hours following intraperitonial lysine injection (Sauer et al. 2011; Posset et al. 2015). The methods used to evaluate lysine degradation metabolites employ mass spectrometric platforms that are based on multiple sample processing protocols and the use of several types of equipment. Typically, PIP is measured by gas chromatography-mass spectrometry (GC–MS) following derivatization of the amine group with methyl chloroformate, acidic ethyl acetate extraction and further derivatization of the carboxyl group to produce the pentafluorobenzyl ester (Kok et al. 1987). SAC, AASA and AAA are usually derivatized with fluorenylmethyloxycarbonyl (FMOC) chloride and analyzed by liquid chromatography tandem mass spectrometry (LC–MS/MS) performed in a triple quadrupole mass spectrometer (Mills et al. 2006; Sadilkova et al. 2009). Alternatively, P6C can be detected and quantified without derivatization before LC–MS/MS analysis (Struys et al. 2012). Since AASA is in equilibrium with P6C, the diagnostic power of measuring either one or the other is comparable (Struys et al. 2012). Here, we propose a LC–MS/MS approach capable of detecting and quantifying all of these metabolites in a single method without derivatization. It was our primary interest to validate the method using a lysine-injected mouse model by monitoring the time course of lysine-derived metabolite levels in peripheral blood.

## Methods

### Chemicals and reagents

Acetonitrile (ACN) was purchased from J.T. Baker (São Paulo, Brazil). Formic acid was obtained from Sigma-Aldrich (São Paulo, Brazil). All solvents used were high performance liquid chromatography (HPLC) grade. Double distilled deionized water (here abbreviated simply as H2O) was obtained from a Milli-Q purification system (Millipore, São Paulo, Brazil). All chemicals and reagents were purchased as of high purity and were stored at appropriate temperatures and conditions. The standard compounds AAA, SAC, PIP, GLU, l-aspartic acid (ASP), l-glutamine (GLN) and pyridoxal-5-phosphate (PLP) were obtained from Sigma-Aldrich (St Louis, MO, USA). AASA/P6C was prepared from allysine ethylene acetal as described (Mills et al. 2006). The internal standards used were the deuterated compounds d3-aminoadipic acid (D3-AAA) and d9-pipecolic acid (D9-PIP), purchased from CDN Isotopes (Pointe-Claire, Quebec, Canada) and ^13^C_4_-Aspatic acid (C13-ASP), purchased from Sigma-Aldrich (St Louis, USA). Stock solutions of 1 mg/mL were prepared for each standard according to manufacturers’ instructions. Standard solutions were stored at −80 °C.

### Analysis of lysine degradation metabolites by LC–MS/MS

#### High-performance liquid chromatography (HPLC)

The chromatographic system consisted of an Agilent 1260 series HPLC with a binary gradient pump. For the analytical separation, a Phenomenex Luna PFP (2) 4.6 × 150.0 mm column with 5.0 µm particles (Alchrom, São Paulo, Brazil) was used. Extracts (10 µL) were injected into the column, and the analytes eluted at a flow rate of 0.8 mL/minute at 30 °C with solvent A (0.1 % formic acid in H_2_O) and solvent B (100 % acetonitrile, ACN). The solvent gradient started with 2 % solvent B (98 % solvent A) for 1.3 min, followed by a linear increase to 60 % B for 7 min, then to 100 % B in 1 min and maintained at this concentration for an additional 2 min followed by the return to the initial conditions. The total run time was 15 min.

#### Mass spectrometry

The analytes were quantified using an ABSciex 5500 Qtrap mass spectrometer coupled with TurboV source (Concord, Ontario, Canada). Analytes were detected using an electrospray ionization detector in positive ion mode set at ion spray voltage of 5.5 kV, curtain gas of 30 psi, collision gas of 9 eV, temperature of 650 °C, nebulizer gas of 70 psi and desolvation gas of 70 psi. For instrument tuning, the analytes were diluted with water containing 0.1 % formic acid and acetonitrile (1:1, v/v) to a final concentration of 50 ng/mL. Analytes were directly infused into the ion source using a Harvard syringe at a flow rate of 10 µL/minute.

Multiple reactions monitoring (MRM) tuning were carried out to optimize fragmentation conditions and identify the best precursor/product transitions for quantitation and confirmation (Additional file 1: Table S1). Scheduled MRM was used with the source parameters tuned using flow injection analysis. The predicted structures of the pairs of precursor and product ions used for quantitation are shown in the Additional file 2: Figure S1.

Both instruments and sources were optimized via the automatic program, available in the software Analyst version 1.6.2. The areas of the detected peaks were integrated using the algorithm MQ4 from the software MultiQuant 3.0.1. The peak areas of the analytes were normalized using those of the corresponding internal standards (added to extracts at final working concentrations of 20 ng/mL).

#### Analytical curves

Calibration curves consisting of nine points per analyte were prepared by two-fold serial dilutions of stock solutions of the internal standards (144, 121 and 145 nM respectively for D9-PIP, D3-AAA and C13-ASP) to achieve the following final range of concentrations: AAA: 1.86 μM–7.27 nM; SAC: 1.085 μM–4.24 nM; PIP: 2.32 μM–9.07 nM; GLU: 2.04 μM–7.96 nM; ASP: 2.25 μM–8.8 nM; PLP: 1.23 μM–4.74 nM and P6C: 2.36 μM–9.22 nM. An artificial plasma solution (Vella et al. 2012) containing 10 % bovine serum albumin (BSA) was used as a surrogate matrix. Six sets of dilutions were prepared independently in the artificial plasma solution and processed as following: mixtures were diluted three times in ACN, followed by vigorously vortexing and centrifugation at 20,000×g for 15 min at 4 °C. The supernatant (ACN extract) was collected and diluted 10 times in H_2_O containing 0.1 % formic acid, and 5 μL were injected for the LC–MS/MS analysis.

#### Animals sampling

Five week old C57BL/6/J female mice were obtained from the Multidisciplinary Center for Biological Investigation on Laboratory Animal Sciences (CEMIB) of the University of Campinas. Experiments were approved by the Ethics Committee of UNICAMP under the protocol number 3625-1. Animals were fasted overnight and then intraperitoneally injected (IP) with 20 mg of lysine diluted in phosphate buffered saline (PBS) or with a solution of 10 mg of pyridoxine hydrochloride in PBS or PBS only as control. Five animals were used per experimental group. For the time-course experiment, approximately 50 μL of peripheral blood was collected by retro-orbital bleeding into EDTA containing tubes at 1, 2, 3, 4 and 6 h post-injection. As a control, blood samples were collected immediately before the lysine injection (0 h). No significant changes were observed for the analytes concentration comparing the 0 h point and PBS injection (data not shown).

#### Metabolite extraction from mouse plasma

Blood was centrifuged at 1500 g for 10 min, and 20 μL of plasma was sampled for metabolite extraction. Proteins were precipitated and metabolites extracted using ACN (2:3), as described in the analytical curves section, containing internal standards to fixed final concentrations (144, 121 and 145 nM respectively for D9-PIP, D3-AAA and C13-ASP). The supernatants containing the polar metabolites were collected, diluted ten times in H_2_O containing 0.1 % formic acid, and used either immediately for LC–MS/MS or stored at −80 °C.

### LC–MS/MS method validation

The LC–MS/MS method was validated according to food and drug administration (FDA) protocols as discussed below (Food Drug Administration Center for Drugs Evaluation Research 2013).

#### Linearity

Calibrations were performed using standards prepared as described previously in the section “analytical curves”. Six replications of the analytical curves were used to obtain the linear equation (y = mx + c).

#### Limits of detection and quantification

Limits of detection (LOD) and limits of quantification (LOQ) were defined as the minimum concentration where the signal was at least 3 times and 10 times higher than the average background noise at the retention time of each analyte, respectively.

#### Accuracy and reproducibility

The accuracy of the method was determined by estimating the data dispersion of individual measures of analytes to multiple aliquots of the matrix (Food Drug Administration Center for Drugs Evaluation Research, 2013). The intra- and inter-day precisions were evaluated at three concentrations: low, median and high (18.8, 37.5 and 75 ng/ml). Five replicates were run at each level. The assay reproducibility was accessed by analyzing three data points in the nanomolar range in triplicate on three different days. The precision determined at each concentration level did not exceed 15 % of the coefficient of variation (CV) except for the LOQ, where it did not exceed 20 % of the CV.

#### Run stability

Stability at the run time was determined by injecting three concentrations in triplicate, directly after sample preparation and after a 12 h overnight experiment. The samples were kept at 10 °C in the auto-sampler.

#### Matrix effect

Matrix effect was calculated by using the equation: B/A * 100, in which B is the analyte corrected area with IS of post-extraction sample and A is the analyte corrected area with IS of external solution, in this case, water was used. Assays were measured in low and high concentration and repeated 3 times (Matuszewski et al. 2003).

#### Selectivity

The selectivity of an analytical method corresponds to the capacity to differentiate and quantify an analyte in the presence of other components in the matrix. The selectivity was obtained using MRM and increased using two mass filters (quantification and confirmation transitions, Additional file 1: Table S1). l-Glutamine (GLN) was included to optimize the chromatographic separation in the hope to avoid interference with the GLU signal, given their similar chemical properties and masses. The PFP(2) column provided satisfactory separation also for this peak pair.

## Results and discussion

### LC–MS/MS method development and validation

**Fig. 2:**
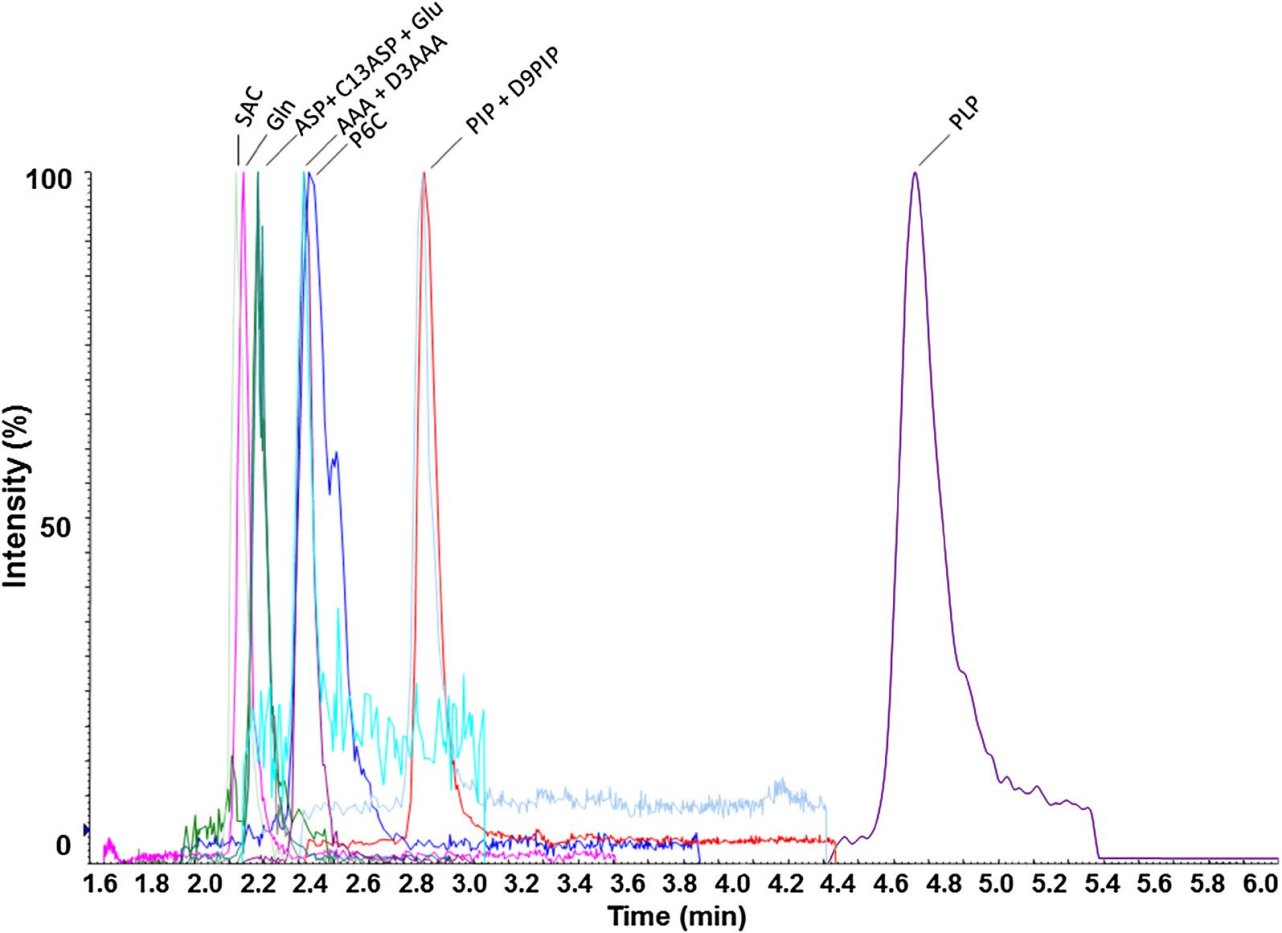
Extracted ion LC–MS/MS chromatogram showing the detection of standard analytes at 37 ng/mL. *SAC* saccharopine, *GLN* glutamine, *ASP* aspartic acid, *C13ASP* ^13^C_4_ labelled aspartic acid, *AAA* aminoadipic acid, *D3AAA* deuterated aminoadipic acid, *P6C* piperideine-6-carboxylic acid, *PIP* pipecolic acid, *D9PIP* deuterated pipecolic acid, *PLP* pyridoxal-5-phosphate

The LC method was developed to retain all polar analytes on the column without the need of derivatization. Satisfactory retention and separation were obtained using the PFP(2) Luna column, composed of pentaflourophenyl groups that can interact with aromatic and polar amino acids (Fig. 2). l-Glutamine (GLN) was included to optimize the chromatographic separation to avoid interference with the GLU signal, given their similar chemical properties and masses. The PFP(2) column provided satisfactory separation also for this peak pair, with a selectivity value of 1.2. PIP and D9-PIP displayed a very subtle change in the retention time (2.87 and 2.85 min, respectively) which is an effect that has been observed in other studies as a common result of the isotopic substitution (Iyer et al. 2004).

The method was validated by analyzing calibration standards in sextuplicate for each unlabeled molecule to determine linear dynamic range, R^2^, retention time, LOD, and LOQ (Table 1). LODs ranged from to 1.6 to 15 nM and LOQs ranged from 6.1 to 25.33 nM. The analysis showed correlations (R^2^) between signal intensity and actual analyte concentrations of >0.99 for all standard curves (Additional file 3: Figure S2). The analytical curves were used to estimate the concentration of each analyte in plasma extracts. Table 2 shows the repeatability and intermediate precision (%RSD), and accuracy for all analytes studied. The lowest %RSD value was 2.6 % for PIP (medium concentration), and the highest %RSD was 15.1 % for GLN (low concentrations). Percent RSD values are in agreement with the accepted values (Boulanger et al. 2005). Accuracy varied from 99.3 % for P6C (low concentration) to 104.5 % for PLP (low concentration). Matrix effects varied from 3.5 % for AAA at low level until 22 % for PLP at low level. These data indicate that the developed LC–MS/MS method allows generating high confidence results. Statistical analysis using one-way ANOVA showed that there were no significant differences between days in which experiments were performed. Molecules were stable for 12 h in the auto-sampler at 10 °C.

**Table 1:** LC-MS/MS method validation parameters

Metabolite	Linear dynamic range (ηM)	Linear dynamic range (ηg mL^−1^)	Linear equation	R^2^	Retention time (min)	LOD (ηM)	LOQ (ηM)
l-Amino adipic acid	7.2–1860	1.17–300	y = 0.0911x + 0.0167	0.995	2.4	1.86	6.20
l-saccharopine	33.9–1085	9.3–300	y = 0.0215x + 0.0042	0.995	2.1	7.60	25.33
l-Pipecolic acid	9.07–2322	1.17–300	y = 0.0366x + 0.0008	0.999	2.9	2.32	7.74
l-Glutamic acid	7.96–2039	1.17–300	y = 0.0789x + 0.0545	0.995	2.2	2.03	6.11
Pyridoxal phosphate	37.9–1213	9.3–300	y = 0.0011x + 0.0020	0.996	4.8	14.97	36.4
Piperideine-6-carboxylic acid	9.2–2359	1.17–300	y = 0.0203x + 0.0053	0.998	2.4	2.35	19.6

The linear equation represents the relation between the area ratio (peak area of analyte divided by peak area of IS) and ηg mL^−1^ concentration

*LOD* limit of detection, *LOQ* limit of quantification

**Table 2:** Method performance as measured in two consecutive days with three different concentrations of metabolites (low, medium and high)

Compound	Theoretical concentration ng mL^−1^	Intraday precision (n = 5) (mean ± S.D.)	Interday precision (n = 10) (mean ± S.D.)	%RSD	%CV
l-Amino adipic acid	18.8	19.1 ± 3.1	19.2 ± 2.2	11.7	99.9
	37.5	36.3 ± 2.1	39.1 ± 4.8	12.4	99.9
	75	75.9 ± 8.47	75.8 ± 6.5	10.5	100.0
l-Saccharopine	18.8	20.3 ± 2.8	19.1 ± 2.5	13.1	99.8
	37.5	38.4 ± 3.8	37.5 ± 4.8	12.7	99.7
	75	71.9 ± 5.1	71.1 ± 5.9	12.3	99.8
l-Pipecolic acid	18.8	19.1 ± 1.4	19.1 ± 0.97	5.1	100.0
	37.5	37.5 ± 0.8	37.4 ± 0.95	2.6	100.1
	75	76.4 ± 8.6	75.1 ± 4.3	5.7	100.0
l-Glutamic acid	18.8	18.7 ± 1.7	18.6 ± 1.6	8.6	100.0
	37.5	35.4 ± 2.1	37.5 ± 3.1	8.3	99.9
	75	72.3 ± 5.7	74.7 ± 5.3	7.1	99.9
l-Glutamine	18.8	20.1 ± 3.8	19.5 ± 2.9	15.1	99.9
	37.5	38.5 ± 4.3	37.6 ± 4.4	11.7	99.7
	75	73.7 ± 5.8	73.7 ± 8.7	8.8	99.9
Pyridoxal phosphate	18.8	18.2 ± 1.5	18.7 ± 1.6	8.4	104.0
	37.5	36.2 ± 1.9	37.9 ± 2.7	7.1	100.0
	75	73.5 ± 6.1	75.7 ± 4.8	6.4	100.1
Piperideine-6-carboxylic acid	18.8	18.5 ± 1.31	19.5 ± 1.4	5.6	99.3
	37.5	36.4 ± 1.2	37.53 ± 0.8	7.8	100.6
	75	74.1 ± 8.6	76.6 ± 5.4	7.9	99.9

The calculated concentrations, %RSD and %CV were obtained using MultiQuant 3.0.1 software (ABSciex)

### Time-course analysis of lysine metabolites in mice plasma

A time-course experiment using 5 week old C57BL/6/J mice was performed with 5 animals per treatment. A PBS solution containing 20 mg of l-lysine, pH 7, was IP injected into each animal, and small aliquots of venous blood were taken at 1 h intervals for 6 h. Control IP injections of PBS solution did not influence the levels of the lysine degradation metabolites (data not shown) and did not differ statistically from the concentrations found at zero time point. Samples were processed for metabolite extraction from plasma and analyzed using the developed LC–MS/MS method.

**Fig. 3:**
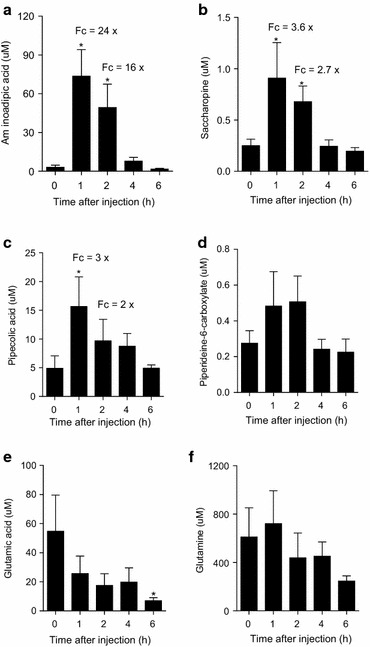
Time-course analysis of lysine degradation metabolites in mouse plasma after IP injection of 20 mg lysine. Fc = fold change as compared to basal concentrations (time 0). *Asterisks* represent statistically significant differences at p < 0.05 (Student’s *T* Test)

The principal metabolite of lysine degradation detected in the mice plasma was AAA, which reached a peak of approximately 70 μM in the plasma 1 h after IP lysine injection (Fig. 3a). This value represents a 24-fold increase compared to the basal AAA values seen at zero time point that was estimated at approximately 3 μM. This value is similar to the 4 μM basal AAA concentration found in normal mouse plasma, as reported in the mouse multiple tissue metabolome database (MMMDB) (Sugimoto et al. 2012) and in the human metabolome database (HMDB 2.0) (Vallat et al. 1996; Wishart et al. 2013) These results suggest that lysine is rapidly oxidized to AAA reaching a maximum within the first 2 h after lysine administration. Four to 6 h following IP lysine injection, AAA returned to concentrations that were not statistically different from that observed for zero time point.

The levels of SAC and PIP (Fig. 3b, c, respectively) followed the same pattern of AAA with highest levels within the first two hours after IP lysine injection. The basal levels of SAC in the mice and human plasma are not listed in the MMMDB or HMDB and thus we could not compare these to the present values. The basal levels of PIP in mice and human plasma as shown in MMMDB (Sugimoto et al. 2012) and HMDB (Baas et al. 2002; Kok et al. 1987) varies from 3.5 to 10.8 μM. These values are in the same range of the PIP concentration (4.9 μM) we found at zero time point. The P6C levels showed similar tendency as the pattern observed for AAA, SAC and PIP but the increased signal detected after 2 h IP lysine injection was not statistically significant (Fig. 3d). The P6C basal levels found in mouse plasma ranged from 0.1 to 0.38 μM, which are similar to the basal levels found in human plasma (Sadilkova et al. 2009). These results suggest that the AASADH enzyme rapidly converts P6C into AAA. It is also possible that the subsequent reaction steps of lysine degradation are slower than the initial steps favoring the accumulation of AAA in the blood within the first 2 h of IP lysine injection.

We also measured the levels of GLU in plasma of the IP lysine injected mice (Fig. 3e) as it is a direct product of saccharopine pathway. GLU is produced by transamination like reaction in with the ε nitrogen of lysine is transferred to α-ketoglutarate during the saccharopine hydrolysis catalyzed by the SDH enzyme. It would be expected that GLU levels would increase after IP lysine injection, but this was not observed. On the other hand, the levels of GLN remained high, and no significant change was observed during the time-course analysis (Fig. 3f). The observed average basal levels of GLU (~55 μM) and GLN (~610 μM) were also within the basal concentration ranges observed in the MMMDB (GLU ~ 25 μM, GLN ~ 400 μM) and the HMDB (GLU ~ 20–100 μM, GLN ~ 490–650 μM).

**Fig. 4:**
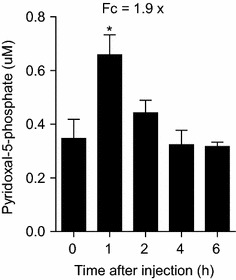
Time-course analysis of pyridoxal-5-phosphate in mouse plasma after IP injection of 10 mg pyridoxine hydrochloride

Absorption of vitamin B6 occurs mainly in the form of pyridoxine, pyridoxal, and pyridoxamine and in the liver these compounds are converted by the enzyme pyridoxal kinase into 5′-phosphate derivatives. Pyridoxamine-5-phosphate and pyridoxine-5-phosphate are then converted to PLP by the enzyme pyridoxamine phosphate oxidase (PNPO) (Mills et al. 2011). We tested IP injection of pyridoxine hydrochloride into mice to determine if increased levels of free PLP are seen at the same time that AAA is produced from lysine catabolism. The levels of free PLP detected during the time-course experiment (Fig. 4) showed that this metabolite was also increased during the first hour after injection, similar to the observed pattern of lysine degradation metabolites.

## Conclusions

We developed a LC–MS/MS method capable of simultaneous detection of the relevant metabolites of lysine catabolism via aminoadipate using a single MS-instrument. The method did not detect AASA but since this compound is in chemical equilibrium with its cyclic form P6C, the detection of the latter is highly correlated to AASA levels (Struys et al. 2012). The quantifications of AAA, SAC, and PIP basal levels were consistent with the basal plasma values listed in the mice MMMDB and human HMDB databases. A time-course analysis of lysine degradation metabolites in mice blood after IP lysine injection revealed that lysine degradation to AAA is a fast process, with a maximum rate occurring within the first 2 h after lysine treatment. This experiment was used to validate the method and, in addition, revealed that lysine degradation occurs at a fast rate, and the levels of catabolites return to basal levels after 4–6 h lysine treatment. The method was also used to detect the levels of PLP after IP injection of pyridoxine. The production of PLP from pyridoxine reached a maximum within 2 h treatment with pyridoxine, similarly to what is observed for the lysine catabolites. This finding may be relevant for PDE since the accumulated P6C may react with PLP just after dietary intake of lysine and pyridoxine. Finally, we suggest that the method developed here which allows simultaneous analysis of lysine degradation metabolites would be useful in investigations of diseases such as PDE, in which this pathway is perturbed.

## Additional files

10.1186/s40064-016-1809-1 MS/MS conditions for analytes and internal standards used for Multiple Reaction Monitoring (MRM). This was the method used for quantitation of metabolites. ChEBI, Chemical Entities of Biological Interest; Q1, Precursor ion; Q3, Fragment product ion; DP, Dispersion potential; CE, Collision energy; CID, Collision-induced dissociation; CXP, collision cell exit potential.
10.1186/s40064-016-1809-1 Predicted pairs of precursor and product ion (shown in left and right of each box, respectively) used for quantitation of the analytes using multiple reaction monitoring (MRM). A, AAA (162 > 98); B, D3-AAA (165 > 101); C, PIP (130 > 84); D, D9-PIP (139 > 93); E, P6C (128 > 82); F, SAC (277 > 130); G, PLP (248 > 150); H, C13-ASP (138 > 76); I, GLU (148 > 84), J, GLN (147 > 130).
10.1186/s40064-016-1809-1 Calibration curves obtained for each analyte in artificial plasma extracts showing the linear correlation between signal (area ratio shown in y axis) and concentration (ng/ml shown x axis). The curve equation and r squared are also shown. The compounds are abbreviated as shown in Table 1.

## Abbreviations

SAC: saccharopine
GLN: glutamine
ASP: aspartic acid
C13ASP: ^13^C_4_-aspartic acid
AAA: aminoadipic acid
D3AAA: deuterated aminoadipic acid
P6C: piperideine-6-carboxylic acid
PIP: pipecolic acid
D9PIP: deuterated pipecolic acid
PLP: pyridoxal-5-phosphate
AASA: α-aminoadipic-δ-semialdehyde
LKR: lysine-ketoglutarate reductase
SDH: saccharopine dehydrogenase
AASS: aminoadipic semialdehyde synthase
AASADH: aminoadipic semialdehyde dehydrogenase
LC–MS/MS: liquid chromatography-tandem mass spectrometry
MRM: multiple reaction monitoring
PFP: pentaflourophenyl
