# Supplementary material for: Simultaneous detection of lysine metabolites by a single LC–MS/MS method: monitoring lysine degradation in mouse plasma
Source: Springerplus. 2016 Feb 25;5:172. doi: 10.1186/s40064-016-1809-1 (PMC4766172; doi:10.1186/s40064-016-1809-1)
Supplement: Supplementary file 1 — 10.1186/s40064-016-1809-1 MS/MS conditions for analytes and internal standards used for Multiple Reaction Monitoring (MRM). This was the method used for quantitation of metabolites. ChEBI, Chemical Entities of Biological Interest; Q1, Precursor ion; Q3, Fragment product ion; DP, Dispersion potential; CE, Collision energy; CID, Collision-induced dissociation; CXP, collision cell exit potential. [file 40064_2016_1809_MOESM1_ESM.docx]

**Table S1:** MS/MS conditions for analytes and internal standards used for Multiple Reaction Monitoring (MRM). This was the method used for quantitation of metabolites. ChEBI, Chemical Entities of Biological Interest; Q1, Precursor ion; Q3, Fragment product ion; DP, Dispersion potential; CE, Collision energy; CID, Collision-induced dissociation; CXP, collision cell exit potential;

| **Analyte** | **Abbreviation** | **ChEBI identifier** | **Q1** | **Q3** | **DP** | **CE** | **CXP** | **Retention Time (min)** |
| --- | --- | --- | --- | --- | --- | --- | --- | --- |
| L-Glutamine | Gln | 18050 | 147.1 | 130 | 41 | 13 | 8 | 2.1 |
|  |  |  | 147.1 | 84 | 46 | 21 | 14 |  |
| L-2-Aminoadipic acid | AAA | 37023 | 162 | 98 | 46 | 21 | 14 | 2.37 |
|  |  |  | 162 | 55 | 46 | 35 | 8 |  |
| DL-2-Aminoadipic-d3 acid | d3-AAA | 37024 (unlabeled) | 165 | 101 | 56 | 21 | 16 | 2.37 |
| DL-Pipecolic-d9 acid | d9-PIP | 17964 (unlabeled) | 139.1 | 93.1 | 71 | 23 | 12 | 2.85 |
| ∆-1-Piperideine-6-carboxylate | P6C | 16987 | 128 | 82 | 101 | 17 | 10 | 2.39 |
|  |  |  |  | 55 | 101 | 27 | 8 | 2.39 |
| L-Pipecolic Acid | PIP | 30913 | 130 | 84.1 | 51 | 21 | 12 | 2.87 |
|  |  |  |  | 56 | 51 | 39 | 8 |  |
| Pyridoxal 5'-phosphate | PLP | 18405 | 248.1 | 150 | 66 | 21 | 10 | 4.85 |
|  |  |  |  | 67 | 66 | 41 | 10 |  |
| L-Saccharopine | SAC | 16927 | 277.1 | 130 | 71 | 21 | 14 | 2.08 |
|  |  |  |  | 84 | 71 | 29 | 14 |  |
| L-Aspartic acid-^13^C_4_ | C13-ASP | 17053 (unlabeled) | 138 | 76 | 56 | 19 | 10 | 2.15 |
| L-Glutamic Acid | GLU | 56-85-9 | 148 | 84.1 | 61 | 19 | 14 | 2.16 |
|  |  |  | 148 | 56 | 61 | 37 | 8 |  |
