# Supplementary material for: Simultaneous detection of lysine metabolites by a single LC–MS/MS method: monitoring lysine degradation in mouse plasma
Source: Springerplus. 2016 Feb 25;5:172. doi: 10.1186/s40064-016-1809-1 (PMC4766172; doi:10.1186/s40064-016-1809-1)

**Figure S1**: Predicted pairs of precursor and product ion (shown in left and right of each box, respectively) used for quantitation of the analytes using multiple reaction monitoring (MRM). A, AAA (162 > 98); B, D3-AAA (165 > 101); C, PIP (130 > 84); D, D9-PIP (139 > 93); E, P6C (128 > 82); F, SAC (277 > 130); G, PLP (248 > 150); H, C13-ASP (138 > 76); I, GLU (148 > 84), J, GLN (147 > 130);


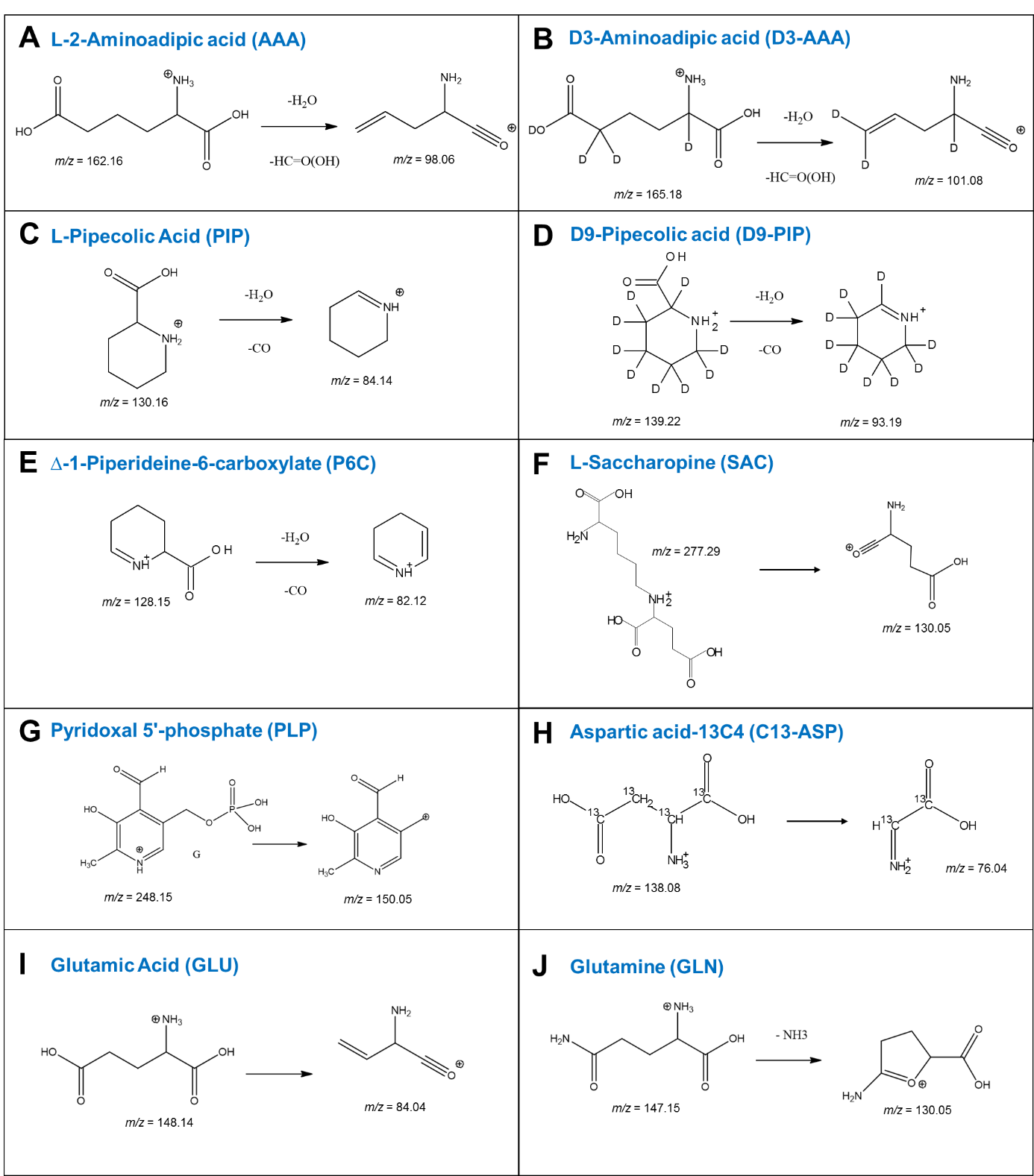

Supplement: Supplementary file 2 — 10.1186/s40064-016-1809-1 Predicted pairs of precursor and product ion (shown in left and right of each box, respectively) used for quantitation of the analytes using multiple reaction monitoring (MRM). A, AAA (162 > 98); B, D3-AAA (165 > 101); C, PIP (130 > 84); D, D9-PIP (139 > 93); E, P6C (128 > 82); F, SAC (277 > 130); G, PLP (248 > 150); H, C13-ASP (138 > 76); I, GLU (148 > 84), J, GLN (147 > 130). [file 40064_2016_1809_MOESM2_ESM.docx]
