# Supplementary material for: Simultaneous detection of lysine metabolites by a single LC–MS/MS method: monitoring lysine degradation in mouse plasma
Source: Springerplus. 2016 Feb 25;5:172. doi: 10.1186/s40064-016-1809-1 (PMC4766172; doi:10.1186/s40064-016-1809-1)
Supplement: Supplementary file 3 — 10.1186/s40064-016-1809-1 Calibration curves obtained for each analyte in artificial plasma extracts showing the linear correlation between signal (area ratio shown in y axis) and concentration (ng/ml shown x axis). The curve equation and r squared are also shown. The compounds are abbreviated as shown in Table 1. [file 40064_2016_1809_MOESM3_ESM.pptx]

## Slide 1
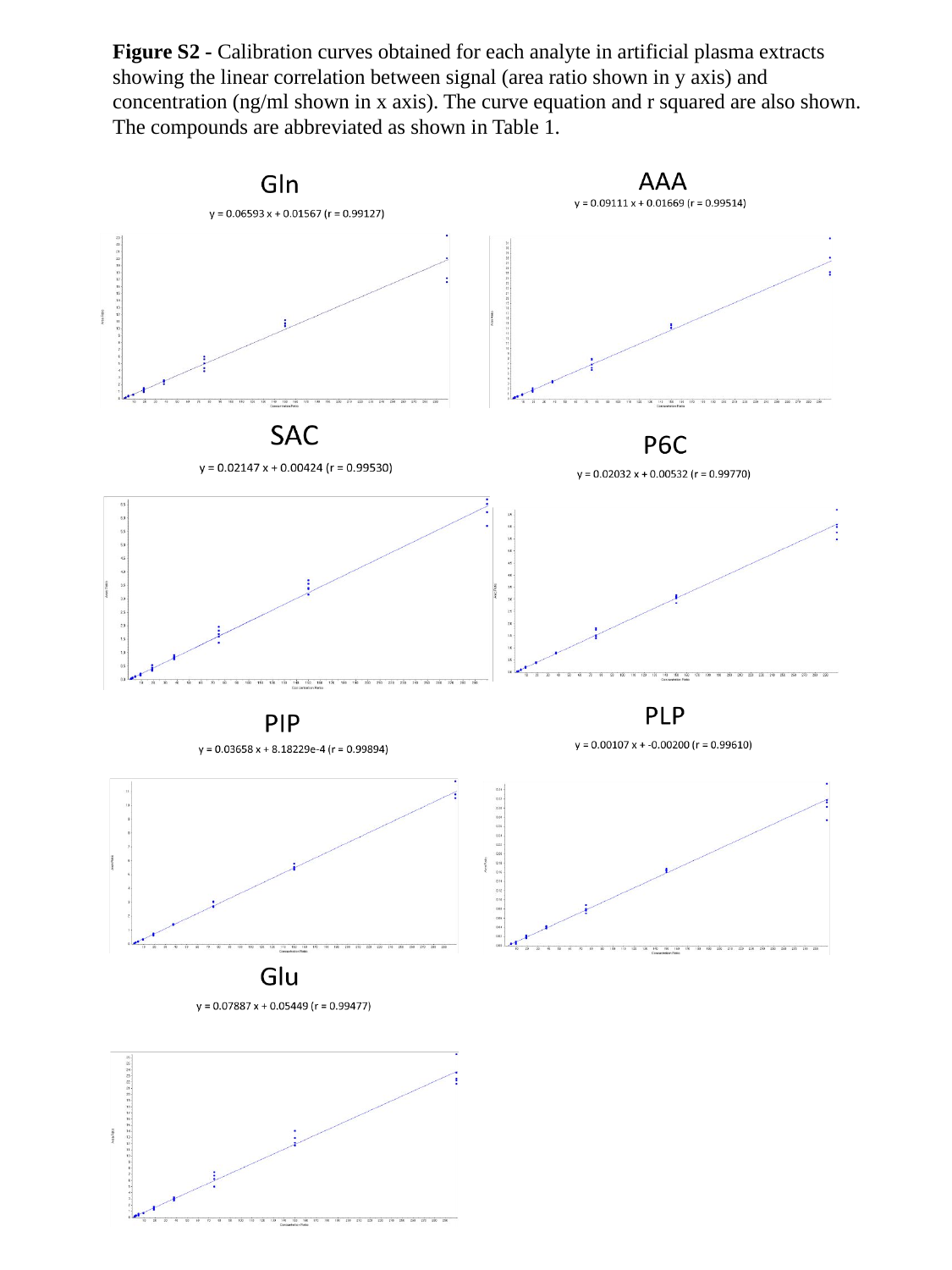

Figure S2 - Calibration curves obtained for each analyte in artificial plasma extracts showing the linear correlation between signal (area ratio shown in y axis) and concentration (ng/ml shown in x axis). The curve equation and r squared are also shown. The compounds are abbreviated as shown in Table 1.
